# Efficacy and safety of chemotherapy as monotherapy in patients with recurrent intermediate/high-risk factors following radical hysterectomy for stage IB-IIA cervical cancer: a single-center retrospective analysis

**DOI:** 10.1186/s12905-024-03135-7

**Published:** 2024-05-18

**Authors:** Bei Dong, Su-Fang Zhou

**Affiliations:** Department of Obstetrics and Gynecology, People’s Hospital of Suzhou High-tech District, No. 95 of Hua-shan Road, High-tech District, Suzhou, 215000 Jiangsu Province China

**Keywords:** Adjuvant chemotherapy, Effectiveness, Radical hysterectomy, Safety, Stage IB-IIA

## Abstract

**Objective:**

The aim of this study is to explore the efficacy and safety of chemotherapy (CT) as a monotherapy in patients with recurrent intermediate/high-risk factors following radical hysterectomy for stage IB-IIA cervical cancer.

**Methods:**

A retrospective analysis was conducted on the medical records of patients diagnosed with stage IB-IIA cervical cancer who underwent radical hysterectomy at the People’s Hospital of Suzhou High-tech District between 2010 and 2020. A total of 66 patients with intermediate or high-risk factors for recurrence were treated exclusively with CT. This cohort included 42 patients in the intermediate-risk group and 24 in the high-risk group. Treatment protocols consisted of 4–6 cycles of paclitaxel and cisplatin drugs for the intermediate-risk group, and 6 cycles for the high-risk group. The relapse-free survival (RFS), recurrence rates, and common CT-related adverse reactions, including bone marrow suppression, nausea and vomiting, and diarrhea, were assessed for both groups.

**Results:**

(1) The cumulative 3-year RFS rates for the intermediate-risk and high-risk groups were 97.3% (36/37) and 82.4% (14/17), respectively, with cumulative 5-year RFS rates of 97.1% (34/35) and 82.4% (14/17), respectively. The Log rank test revealed no significant difference between the two groups (*P* > 0.05), (*χ*² = 2.718, *P* = 0.099). The 5-year recurrence rates in the intermediate-risk and high-risk groups were 2.38% (1/42) and 12.50% (3/24), respectively. (2) The incidence of grade III bone marrow suppression in the intermediate-risk and high-risk groups was 21.19% (11/42) and 25.00% (6/24), respectively, while the incidence of grade IV bone marrow suppression was 11.90% (5/42) and 8.33% (2/24), respectively. There was no statistically significant difference in bone marrow suppression grades between the two groups (*P* > 0.05).

**Conclusion:**

CT with paclitaxel and cisplatin, administered as monotherapy post-radical hysterectomy for stage IB-IIA cervical cancer, demonstrates satisfactory survival benefits with an acceptable safety profile. Moreover, no significant differences were observed in prognosis or adverse reactions between the different risk groups treated solely with CT.

## Introduction

Cervical cancer represents the most prevalent malignant tumor of the female reproductive system. In 2018, there were over 569,000 new cases of cervical cancer worldwide, with 85% of the cases originating in developing countries [1, 2]. Currently, early-stage cervical cancer treated with radiotherapy (RT) or radical hysterectomy + pelvic lymph node dissection can achieve 5-year survival rates of 80–90% [3]. However, the presence of high-risk factors such as positive surgical margins, lymph node metastasis, and parametrial invasion, as well as intermediate-risk factors including tumor size, depth of stromal invasion (DOI) and/or lymphovascular space invasion (LVSI), [4, 5] often necessitates the application of postoperative RT or concurrent chemoradiotherapy (CCRT) to reduce the rates of local or distant recurrence. Despite these measures, local recurrence occurs in 20–30% of patients, and 18–25% develop distant metastasis [6, 7]. Moreover RT is frequently associated with severe gastrointestinal and genitourinary symptoms [8, 9], and is constrained by its dose limits and the infeasibility of addressing subclinical symptoms beyond the radiation field due to the proximity of peripelvic organs. Consequently, current research priorities include the evaluation of the efficacy and safety of chemotherapy (CT) as a monotherapy following radical hysterectomy in patients with stage IB-IIA cervical cancer who present with recurrent intermediate/high-risk factors, which pose significant clinical challenges.

## Materials and methods

### General information

In this retrospective study, patients with stage IB-IIA cervical cancer who underwent radical hysterectomy and pelvic lymph node dissection, with or without abdominal aortic lymph node sampling, at our hospital from 2010 to 2020 were considered for inclusion.

To qualify for the study, participants needed to meet all of the following criteria: (1) Radical resection of stage IB - IIA cervical cancer. (2) All surgical procedures were performed at our hospital, involving type III extensive resection of paravaginal and uterine tissues along with pelvic lymph nodes. (3) Postoperative pathological assessments indicated the presence of high or intermediate risk factors for recurrence. (4) Patients did not receive neoadjuvant CT or RT prior to surgery. (5) Only postoperative CT was administered. (6) Patient information was complete and adequately recorded. Exclusion criteria encompassed failure to meet any of these conditions.

Ultimately, 66 patients were included in the analysis, with 42 (63.6%) classified in the intermediate-risk group and 24 (36.4%) in the high-risk group. Patient demographics and clinical characteristics are detailed in Table 1.

**Table 1:** Patient demographics

	Intermediate-risk group (*n* = 42)	High-risk group (*n* = 24)
Average age (years)	51.048 ± 7.525	49.125 ± 9.013
Clinical stage (n)		
IB1	26	6
IB2	6	8
IIA	10	10
Pathological type (n)		
Squamous carcinoma	38	18
Adenocarcinoma + Adenosquamous carcinoma	4	6
Differentiation degree (n)		
Highly differentiated (G1)	4	0
Moderately differentiated (G2)	22	16
Poorly differentiated (G3)	16	8
Cervical invasion (n)		
≤ 1/2 cervical thickness	22	8
> 1/2 cervical thickness	20	16
LVSI (n)		
No	22	10
Yes	20	14
Pelvic lymph node involvement (n)		
No	42	6
Yes	0	18
Parametrial invasion (n)		
No	42	6
Yes	0	18
Specimen incisal margin (n)		
Negative	42	22
Positive	0	2

High-risk factors for recurrence included at least one of the following three criteria: (1) Positive lymph nodes; (2) Parametrial involvement in tumor invasion; (3) Positive surgical margins. Intermediate-risk factors for recurrence referred to the absence of high-risk factors and the fulfillment of any of the following four criteria: (1) Maximum tumor diameter ≥ 4 cm; (2) Tumor invasion of > 1/2 cervical thickness; (3) Poorly differentiated tumor; (4) Presence of LVSI.

### Treatment methods

#### Surgery

The primary intervention comprised an extensive total hysterectomy via laparotomy/laparoscopy, accompanied by pelvic lymph node dissection. This was conducted with or without abdominal aortic lymph node sampling and included bilateral or unilateral adnexectomy where deemed necessary.

### Postoperative CT

Following surgery, all 66 patients underwent CT exclusively. The regimens for cervical squamous cell carcinoma, adenocarcinoma, and adenosquamous carcinoma included paclitaxel and cisplatin. Paclitaxel was administered intravenously at a dosage of 175 mg/m^2^ over a 3-hour period on the first day. On the second day, cisplatin was administered intravenously at a dosage of 50 mg/m^2^ over 60 min. The treatment cycle lasted for 3 weeks. The intermediate-risk group received 4 to 6 cycles, while the high-risk group received 6 cycles. The CT-related adverse reactions were assessed using the WHO criteria for grading adverse reactions in cancer therapy, ranging from 0 (no change) to IV (extreme toxicity) [10].

### Follow-up and statistical methods

All the patients were followed up according to the cervical cancer follow-up standards, with follow-ups conducted until December 2022. The follow-up duration ranged from 17 to 146 months, with a median follow-up period of 94 months. Statistical analysis was performed using the IBM SPSS (Statistical Product and Service Solutions) 22.0 software. The Kaplan–Meier and log rank methods were used to calculate and compare the cumulative relapse-free survival (RFS) rate between the two groups, and the chi-squared test was used for comparing enumeration data.

## Results

### RFS and recurrence

**Fig. 1:**
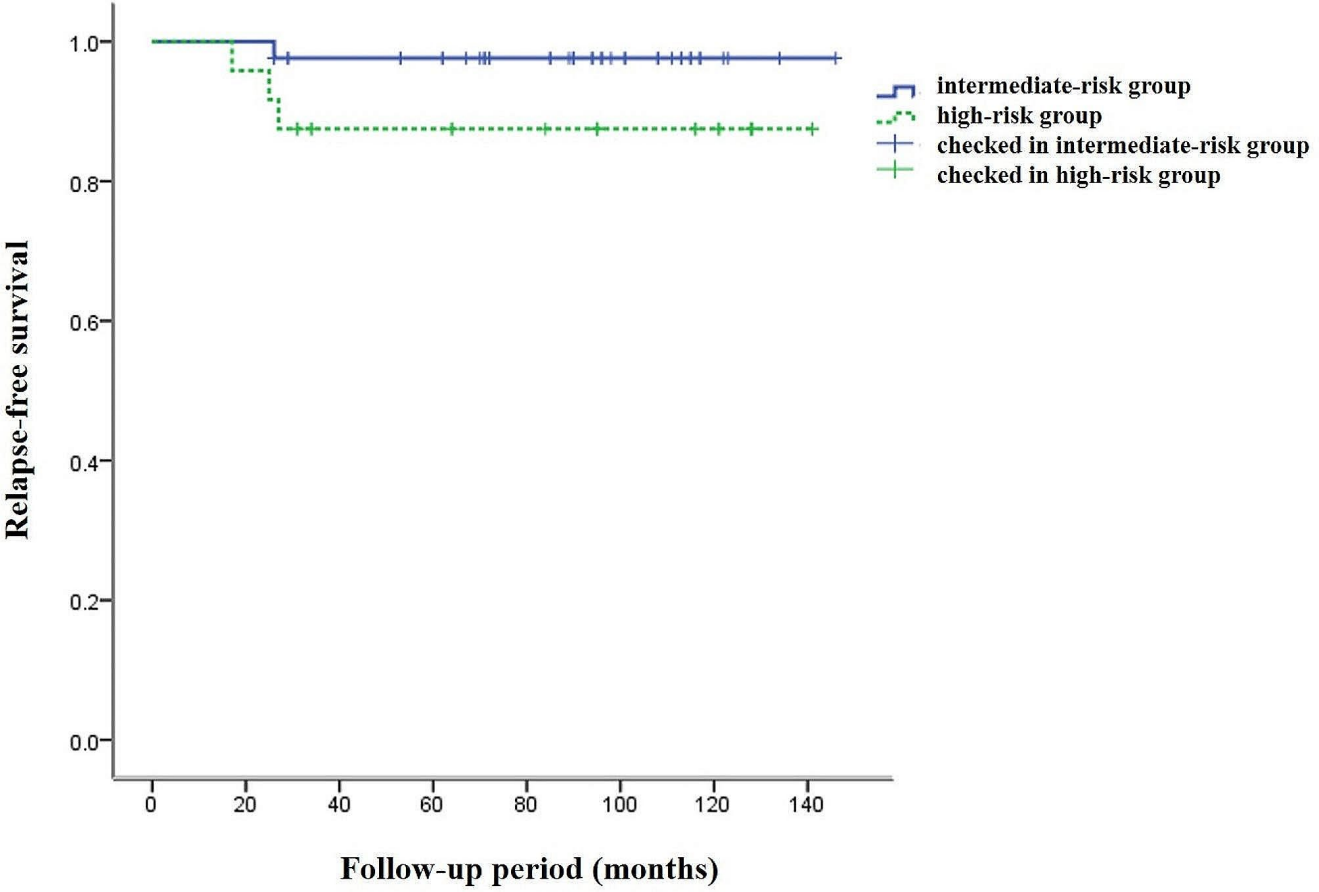
Two sets of survival functions

In the intermediate-risk and high-risk groups, the cumulative 3-year RFS rates were 97.3% (36/37) and 82.4% (14/17), respectively, and the cumulative 5-year RFS rates were 97.1% (34/35) and 82.4% (14/17), respectively. According to the log rank test, there was no statistically significant difference between the two groups (*P* > 0.05, *χ*² = 2.718, *P* = 0.099). The survival functions for both groups are shown in Fig. 1. Among the 42 patients in the intermediate-risk group, 1 patient experienced pelvic recurrence within 5 years, resulting in a recurrence rate of 2.38% (1/42). This patient, diagnosed with stage IB1 squamous carcinoma grade 3, exhibiting more than half cervical thickness invasion, and LVSI, developed pelvic recurrence 26 months postoperatively and received RT at another facility; she remains alive with the disease.

In the high-risk group, 3 out of 24 patients experienced recurrence within 5 years, 2 experienced local recurrence (8.33%, 2/24) and 1 experienced distal recurrence (4.17%, 1/24), resulting in a recurrence rate of 12.50% (3/24). The first patient, with stage IIA grade 2 cervical adenocarcinomas, demonstrated more than half cervical thickness invasion, LVSI, pelvic lymph node metastasis, and parametrial invasion, and experienced pelvic recurrence 17 months post-surgery. She received RT at another facility and is currently living with the disease. The second patient, diagnosed with stage IIA grade 2 cervical adenosquamous carcinomas, had similar risk factors and developed pelvic recurrence 27 months after surgery. She underwent additional CT and died two years post-recurrence. The third patient, with stage IB1 grade 2 cervical adenocarcinoma, presented with over half cervical thickness invasion, LVSI, pelvic lymph node metastasis, parametrial invasion, and positive surgical margins. She developed pelvic recurrence and bone metastasis 25 months postoperatively and underwent RT and CT at another facility; she died one year following the recurrence. Over a five-year postoperative period, 4 out of the 66 patients experienced recurrences, leading to an overall recurrence rate of 6.06% (4/66), encompassing 3 pelvic and 1 extrapelvic recurrence, as detailed in Table 2.

**Table 2:** Clinicopathological characteristics of the patients with recurrence

Group	Patients	Age of onset (years)	Stage	Pathological type	Intermediate/high-risk factors	Recurrence site	Time to recurrence (months)	Post-relapse treatment	Outcome
Intermediate-risk group	Case 1	59	IB1	Squamous carcinoma	G3, tumor invasion of > 1/2 cervical thickness, LVSI	Pelvic cavity	26	RT	Survival with tumor
High-risk group	Case 1	48	IIA	Adenocarcinoma	G2, tumor invasion > 1/2 cervical thickness, LVSI, pelvic lymph node metastasis, parametrial invasion	Pelvic cavity	17	RT	Survival with tumor
	Case 2	71	IIA	Adenosquamous carcinoma	G2, tumor invasion > 1/2 cervical thickness, LVSI, pelvic lymph node metastasis, parametrial invasion	Pelvic cavity	27	CT	Death
	Case 3	58	IB1	Adenocarcinoma	G2, tumor invasion > 1/2 cervical thickness, LVSI, pelvic lymph node metastasis, parametrial invasion, positive surgical margins	Pelvic cavity + bone	25	RT + CT	Death

Note: LVSI, lymphovascular space invasion; CT, chemotherapy; RT, radiotherapy

### CT toxicity

#### Bone marrow suppression

In the intermediate-risk group, 26 patients experienced grade I-II bone marrow suppression, with an incidence rate of 61.90% (26/42), 11 patients experienced grade III bone marrow suppression, with an incidence rate of 21.19% (11/42), and 5 patients experienced grade IV bone marrow suppression, with an incidence rate of 11.90% (5/42). In the high-risk group, 16 patients experienced grade I-II bone marrow suppression, with an incidence rate of 66.67% (16/24), 6 patients experienced grade III bone marrow suppression, with an incidence rate of 25.00% (6/24), and 2 patients experienced grade IV bone marrow suppression, with an incidence rate of 8.33% (2/24). There was no statistically significant difference in the degree of bone marrow suppression between the two groups (*P* > 0.05), as shown in Table 3.

**Table 3:** Comparison of bone marrow suppression grades between low and high-risk groups

	Bone marrow suppression grade		
Group	I-II	III	IV
Intermediate-risk, n	26	11	5
High-risk, n	16	6	2
*χ*²	1.556	0.011	-
*P*	0.884	0.915	0.498

Note: *P*<0.05 indicates statistically significant difference

During the treatment, patients experiencing bone marrow suppression were administered recombinant human granulocyte colony-stimulating factor (rhG-CSF), which facilitated their recovery to normal levels without resulting in adverse outcomes. For those who experienced grade IV bone marrow suppression, isolation in a dedicated ward, enhanced personal protection protocols, and environmental disinfection were implemented. These patients successfully passed through the critical period without complications. As shown in Table 3, both groups exhibited decreases in white blood cells (66/66, 100%) and neutrophils (65/66, 98.5%). The incidence of thrombocytopenia was 28.8% (19/66); all cases were mild, did not require special treatment, and gradually normalized alongside the recovery of white blood cells and neutrophils. The incidence of anemia was 62.1% (41/66), with mild cases accounting for 54.5% (36/66) and moderate cases for 7.6% (5/66). Treatment with oral iron succinate was administered, leading to a gradual normalization of hemoglobin levels.

Importantly, there were no instances of treatment discontinuation or severe adverse events attributable to significant bone marrow suppression in either patient group.

### Nausea and vomiting

In the intermediate-risk group, 95% (38/42) of the patients experienced grade I-II nausea and vomiting, while 4.8% (2/42) experienced grade III nausea and vomiting. In the high-risk group, 41.7% (10/24) experienced grade I-II nausea and vomiting, with no grade III-IV events. Management of these symptoms was effectively conducted using ondansetron, a 5-HT3 receptor antagonist, administered both intravenously and orally. These side effects did not lead to any discontinuation or alteration of CT drugs.

### Diarrhea

In the intermediate-risk group, 9.5% (4/42) of the patients experienced grade I-II diarrhea, while in the high-risk group, 8.3% (2/24) experienced grade I-II diarrhea. There were no grade III-IV events in either group. Patients who developed diarrhea were advised on dietary modifications and were administered montmorillonite powder orally as needed. None of the patients discontinued treatment or changed CT drugs due to diarrhea.

Details of adverse reactions in both groups are shown in Table 4.

**Table 4:** Safety assessment

	Intermediate-risk group			High-risk group			Total
Grade	I-II	III	IV	I-II	III	IV	
Leukopenia, n (%)	26 (61.9)	11 (26.2)	5 (11.9)	16 (66.7)	6 (25.0)	2 (8.3)	66 (100)
Neutropenia, n (%)	24 (57.1)	12 (28.6)	5 (11.9)	15 (62.5)	7 (29.2)	2 (8.3)	65 (98.5)
Thrombocytopenia, n (%)	10 (23.8)	0	0	9 (37.5)	0	0	19 (28.8)
Anemia, n (%)	23 (54.8)	3 (7.14)	0	13 (54.2)	2 (8.3)	0	41 (62.1)
Nausea and vomiting, n (%)	38 (90.5)	2 (4.8)	0	10 (41.7)	0	0	50 (75.8)
Diarrhea, n (%)	4 (9.5)	0	0	2 (8.3)	0	0	6 (9.1)

## Discussion

For patients undergoing surgical treatment for cervical cancer, adjuvant therapy is tailored based on the recurrence risk factors identified postoperatively. CCRT is the standard adjuvant therapy for those with high-risk factors. In cases where clinicopathological assessments reveal a combination of intermediate-risk factors, postoperative RT is recommended [11]. The primary objective of adjuvant therapy extends beyond controlling local recurrence to also aiming at reducing the risk of distant metastases. However, while adjuvant pelvic radiation may lower the incidence of local recurrences [12], it is not effective in preventing distant metastases [13], which contributes to the limited impact of postoperative RT on overall survival [14]. 

Moreover, adjuvant RT escalates the risk and exacerbates the severity of certain surgical complications, such as urinary fistulae and lower limb lymphedema [15]. Additionally, the combination of surgery and RT can lead to a diverse range of complications, increasing the likelihood of bladder and bowel dysfunction, sexual and psychological issues, and significantly impairing the quality of life [15, 16]. In contrast, CT might offer benefits in terms of fewer treatment-related complications and potentially improve postoperative quality of life by avoiding radiation-induced morbidity.

Given the limited long-term survival advantage offered by RT and the heightened risk of adverse events, adjuvant CT has been proposed as an alternative. Several studies have been conducted to assess the efficacy and safety of adjuvant CT alone in patients with high or intermediate-risk factors following radical surgery in early-stage cervical cancer.

### CT for patients with recurrent high-risk factors

Matoda et al. [17] conducted a study on 62 patients with stage IB-IIA cervical cancer who underwent radical hysterectomy and had pelvic lymph node metastasis. These patients received CT as a monotherapy using the irinotecan combined with nedaplatin protocol. The treatment started within 6 weeks after the surgery and was repeated every 28 days, for a maximum of 5 cycles. Among the study participants, 55 patients (88.7%) completed the planned treatment cycles. The 2-year and 5-year RFS rates were 87.1% and 77.2%, respectively. During the follow-up period, 14 patients (22.5%) experienced recurrence, with 8 deaths. The 5-year overall survival (OS) rate was 86.5%, and only 9.7% of the patients experienced leg lymphedema. No patient died of treatment-related complications. This suggests that CT has good and long-lasting efficacy and safety. Distant metastasis remains a significant challenge in the treatment of high-risk cervical cancer when RT alone is employed. CT is regarded as a more effective strategy for eradicating subclinical distant metastases [18]. Iwasaka et al. [19] conducted a study comparing the efficacy of adjuvant CT to that of adjuvant RT in patients with recurrent high-risk factors following radical hysterectomy. The findings revealed that intra- and extrapelvic recurrences in the CT group constituted 85% and 23% of all recurrences, respectively, whereas the RT group exhibited recurrences at rates of 38% intra-pelvic and 71% extra-pelvic (*P* < 0.01). This suggests that adjuvant CT can significantly reduce extrapelvic recurrences. Additionally, the study highlighted the benefits of CT in managing local failures, with side effects reported to be within acceptable limits.

In 2019, Akiko et al. [20] launched a phase III clinical trial aimed at determining whether postoperative adjuvant CT could match the effectiveness of adjuvant concurrent CT in terms of overall survival for patients with high-risk cervical cancer. This study is currently ongoing.

### CT for patients with recurrent intermediate-risk factors

Currently, the guidelines for managing early-stage cervical cancer patients with intermediate-risk factors vary by region, and the research on postoperative adjuvant therapies is inconsistent [21]. A growing body of retrospective studies questions the role of adjuvant radiation following radical surgery for patients with intermediate-risk factors [22–26], shifting focus towards the potential benefits of adjuvant CT.

Takeshima et al. [18] reported a 5-year disease-free survival (DFS) rate of 93.3% in patients with intermediate-risk tumors, noting that the side effects from CT were acceptable. Similarly, Lee et al. [27] conducted a retrospective analysis of 76 stage IB-IIA patients with a combination of 3 intermediate-risk factors without LN metastasis in South Korea who underwent cervical cancer radical surgery followed by adjuvant CT alone. The study found a 3-year DFS rate of 94.6%, a 5-year overall survival (OS) rate of 90.6%, and a 5-year disease-specific survival rate of 96.2%, suggesting that CT alone may be a viable adjuvant option for patients with surgical-pathologic risk factors. Lee et al. [28] compared outcomes between adjuvant CT and RT for patients with surgically confirmed intermediate risk factors in stage IB-IIA cervical cancer, finding no significant difference in DFS rates. A nationwide study of 555 patients with stage IB cervical cancer with recurrent intermediate-risk factors showed that DFS and cause-specific survival rates for those who received CT were comparable to those who underwent CCRT, with similar risks for local and distant recurrences [29]. 

Nie et al. [30] retrospectively analyzed clinicopathologic data of 596 patients diagnosed with stage I-IIA cervical cancer at the Obstetrics and Gynecology Hospital of Fudan University from January 2013 to November 2015. Among these patients, 500 received CT, RT, sequential CT, or combined CT and RT. The 5-year progression-free survival (PFS) and the OS rate for the entire cohort were 90.4% and 90.9%, respectively. Compared with the patients in the control group, those receiving CT, RT, or combined CT and RT showed improvements in the PFS and OS (*P* < 0.05) rates. The RT group had lower PFS and OS than the CT and combined CT and RT groups (*P* < 0.05). The study found that patients receiving CT, either alone or combined with RT, exhibited improved PFS and OS rates compared to those in the control group and significantly better outcomes than those receiving only RT.

In 2018, Lee et al. [31] conducted a meta-analysis comparing the efficacy of adjuvant CT and adjuvant RT/CT in patients with cervical cancer patients with recurrent high/intermediate-risk factors post-radical surgery. The results indicated no statistically significant differences in recurrence and mortality rates between the groups, with adjuvant CT showing a trend towards reducing the risk of distant metastasis.

### Strengths and implications

Traditionally, CT was not considered effective for cervical cancer. However, with the growing recognition of the side effects associated with postoperative RT, the role of CT in managing early-stage cervical cancer with post-surgical recurrence risk factors has emerged as a focal point of research. In this retrospective study, we assessed the safety and efficacy of CT in patients with moderate to high risk factors for recurrence, as confirmed pathologically after radical surgery for stage IB-IIA cervical cancer over a 10-year period at our hospital.

The intermediate-risk group exhibited cumulative 3-year and 5-year RFS rates of 97.3% and 97.1%, respectively. Meanwhile, the high-risk group showed cumulative 3-year and 5-year RFS rates of 82.4%. Notably, there were no cases of distal recurrence in the intermediate-risk group, and only one patient (4.17%) in the high-risk group experienced distal recurrence. Despite the absence of pelvic RT, the local recurrence rates were 2.38% in the intermediate-risk group and 8.33% in the high-risk group. All patients demonstrated acceptable drug safety and tolerability, with no treatment discontinuations or changes in CT drugs due to severe adverse events.

These findings align with prior research and suggest that adjuvant CT alone may be sufficient to prevent both local and distal recurrences after complete radical surgery. Although our study is limited by the absence of a control group and its small sample size, it contributes valuable insights that may guide further research. The potential future validation of CT’s effectiveness could expand treatment options, particularly in regions with limited medical resources or for patients who are unsuitable for RT due to conditions like pelvic adhesive disease, intraoperative abdomino-pelvic injuries, radiation intolerance, or non-compliance with radiation treatment schedules.

However, the potential systemic adverse reactions associated with CT necessitate thorough patient evaluations prior to its administration. Active management of adverse reactions through intensive monitoring during and after treatment is crucial. Should serious adverse reactions occur, CT may need to be discontinued and alternative therapeutic options considered based on the patient’s condition.

### Limitations

This study faces several limitations that impact the generalizability and robustness of its conclusions. Firstly, as a retrospective analysis conducted at a single institution, the applicability of the findings to broader populations is limited. Secondly, the absence of a comparative study with groups receiving RT and CCRT means that the effectiveness and safety of CT relative to these treatments remain incompletely characterized. Further research is necessary to provide a more comprehensive evaluation. Additionally, the small sample size of this study restricts our ability to detect finer differences in survival outcomes, particularly in subgroup analyses that might require comparing different surgical techniques or other specific variables. A substantially larger sample size would be necessary to conduct such detailed analyses effectively. Moreover, the retrospective nature of the study led to incomplete data on complications, further constraining the study’s findings.

## Conclusion

For patients with stage IB-IIA cervical cancer who have undergone radical hysterectomy and present with recurrent intermediate/high-risk factors based on pathological assessment, administering standardized CT as a monotherapy appears to offer satisfactory survival benefits and acceptable safety profiles. The results indicate no significant differences in prognosis and adverse reactions among different risk groups, suggesting that CT alone could expand treatment options for patients requiring further intervention post-surgery. However due to the limited sample size and the absence of a control group, further studies are required to assess the superiority of CT compared to RT and CCRT.

## Data Availability

All data generated or analysed during this study are included in this article. Further enquiries can be directed to the corresponding author.
